# Supplementary material for: Nonlinear synchronization through vector subharmonic entrainment
Source: Commun Phys. 2026 Jan 21;9(1):71. doi: 10.1038/s42005-026-02509-7 (PMC12923357; doi:10.1038/s42005-026-02509-7)
Supplement: Supplementary file 2 — Supplementary Information [file 42005_2026_2509_MOESM2_ESM.pdf]

Supplementary Information for Nonlinear synchronization through vector subharmonic entrainment

Dmitrii Stoliarov[1]*†, Sergey Sergeyev[1]†, Hani Kbashi[1], Fan Wu[2], Qianqian Huang[2], Chengbo Mou[2]*†

[1]*Aston Institute of Photonics Technologies, Aston University, Birmingham, B4 7ET, UK.

[2]Key Lab of Specialty Fiber Optics and Optical Access Network, Shanghai University, , Shanghai, 200444, China.

*Corresponding author(s). E-mail(s): d.stoliarov@aston.ac.uk; mouc1@shu.edu.cn

Contributing authors: s.sergeyev@aston.ac.uk ; h.kbashi@aston.ac.uk; fanwu@shu.edu.cn ; huangqq@shu.edu.cn ;

†These authors contributed equally to this work.

Supplementary Note 1: Description of the experimental setup

The detailed schematic of the experimental setup is shown in Fig. S1.

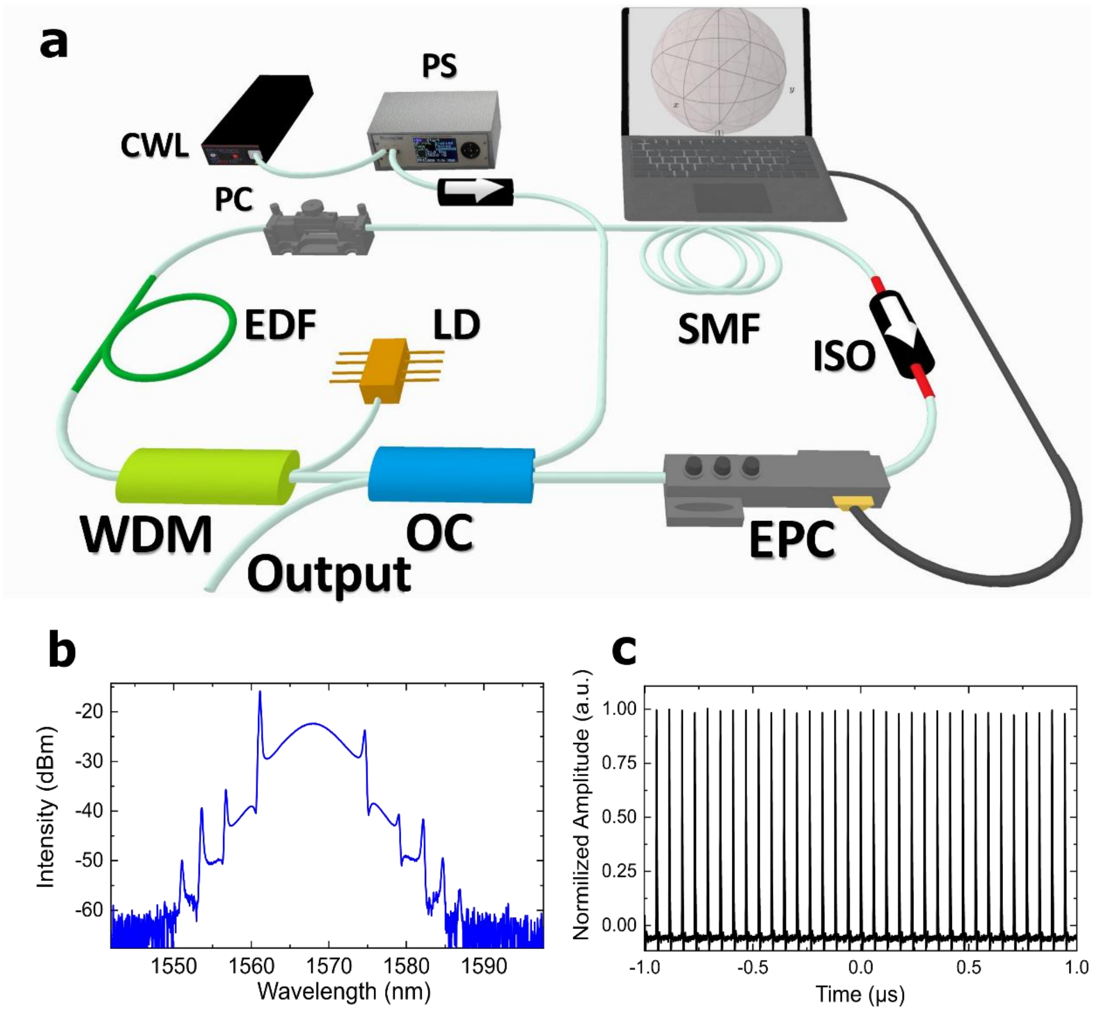

Fig. S1 Assembled mode-locked Er fiber laser. a) Schematic setup of the NPR mode-locked fibre laser. EDF: erbium-doped fiber; LD: 980 nm pump laser diode; PC: manual polarization controller, EPC: electronically driven polarization controller, ISO and ISO IN: polarization sensitive isolators; WDM: wavelength division multiplexer; PS: polarization scrambler; CWL: continuous wave laser, OC: 70/30 optical coupler; b) Output spectra and (c) oscilloscope trace of the Er-fibre laser setup operating in mode-locked regime.

The system utilizes a short piece of highly erbium-doped, single-mode optical erbium-doped fibre (EDF) with a peak core absorption of around 110 dB/m at 1530 nm, cutoff wavelength of 890 nm, the numerical aperture of 0.2. The EDF is pumped by a 976-nm pigtailed laser diode (LD) through a 980/1550 nm wavelength division multiplexer (WDM). The EDF is subsequently spliced to the output port of a dual-stage polarization-sensitive optical isolator (ISO) with a centre wavelength of 1550 nm and an extinction ratio of 28 dB. The 70/30 optical coupler (OC), featuring a coupling ratio centred at 1550 nm and an insertion loss around 1 dB, was employed to extract 30% of the laser power from the cavity. In addition, the configuration includes two fibre polarization controllers placed before and after the polarization-dependent optical isolator. The first one is the in-line miniature manual in-line polarization controller (PC). The second one is the electronic in-line polarization controller (EPC) with a three-section external voltage control. The EPC software enables full state-of-polarization control through thermal technology, ensuring stable polarization scanning across the entire Poincaré sphere. It is important to note that, except for the ISO and EDF, the full cavity of the laser was constructed using standard fibre SMF-28. The total fibre laser cavity length is 12.3 m comprising 0.2 m EDF, 1.5 m PM-1550 fibre, and 10.5 m of SMF-28 fibre. This length also includes pigtail fibres from the Electronic Polarization Controller (EPC), isolator, and coupler. The anomalous group velocity dispersion (GVD) values of the SMF-28 and PM-1550 fibre at 1.55 μm were about -23 ps^2/km and -22 ps^2/km, respectively. The normal GVD of the Er-doped fibre at 1550 nm was estimated as 15.3 ps^2/km. As a result, the net dispersion of the cavity could be calculated as -0.27 ps^2, suggesting that the laser was operating at an anomalous dispersion regime. For optical signal injection, a Polarization Scrambler (PS) EPS1000 (Novoplet) and a narrow linewidth continuous wave laser (CWL) CoBrite DX1 (IDPhotonics) with tunable wavelength are employed. The setup seeds an optical power of 15 dBm at 1.67 kHz through a vacant input port of a 30/70 output optical coupler. The output light pulses were characterized and analysed using various detection and measurement techniques, including a 50 GHz High-Speed Photodetector, a real-time oscilloscope with 6 GHz bandwidth, and an optical spectrum

analyser AQ6317B (Yokogawa). A fast polarimeter PM1000-XL-FA-N20 D (Novoptel) with a sampling frequency of 100 MS/s to detect the evolution of the Stoke parameters S_1, S_2, S_3, and the total power S_0. The stable mode-locked regime was achieved by adjusting the polarization controllers in the absence of CWL injection. Figures 1b and c demonstrate the optical spectra and oscilloscope trace of the stable single-pulse mode-locked regime with a fundamental pulse repetition rate of 16.67 MHz.

Supplementary Note 2: Linear stability analysis.

By substituting $u = |u|exp(i \cdot \varphi_x)$ and $v = |v|exp(i \cdot \varphi_y)$ into Eqs. 6-10, we find the steady-state solution for the case of absence of the CW signal in the cavity, e. g. $a = 0$ and $(du/dt = dv/dt = df_1/dt = df_2/dt = df_2/dt = df_3/dt \equiv 0)$ as $|u| = |v| = E_s$; $\Delta\varphi = \varphi_y - \varphi_x = 0, \pi$; $f_{2s} = 0$ from the equations:

$$\frac{\alpha_1}{1+\Delta^2} f_{1s} - \alpha_2 + A + \left(\frac{\alpha_1}{1+\Delta^2} f_{3s} + A\right) \cdot m = 0, a_1 f_{1s} + a_3 f_{3s} = b_1,$$
$$\frac{a_3}{2} f_{1s} + a_1 f_{3s} = 0. \qquad \text{(S1)}$$

Here

$$b_1 = \frac{\chi-1}{2} I_p - 1, a_1 = 1 + \frac{I_p}{2} + \frac{2\chi E_s^2}{1+\Delta^2}, a_3 = \frac{2\chi E_s^2 \cdot m}{1+\Delta^2},$$
$$A_{11} = A_{22} = -1.1543,\ A_{12} = A_{21} \approx 1.1543, m = \pm 1. \qquad \text{(S2)}$$

The results for E_s, f_{1s}, f_{3s} for $m = \pm 1$ and $\alpha_1 = 10.131, \varepsilon = 0.6 \cdot 10^{-5}, \alpha_2 = 2.3, \Delta = 0.015,\ \chi = 2.3, \gamma = 2 \cdot 10^{-6}, \delta = 1$ and $a = 0$ are shown in Fig. 6 (a).

For linear stability analysis, we substitute the following ansatz into Eqs. (6-10):

$$\boldsymbol{F}(t) \equiv \begin{pmatrix} |u| \\ |v| \\ \Delta\varphi \\ f_1 \\ f_2 \\ f_3 \end{pmatrix} = \begin{pmatrix} E_s \\ E_s \\ 0\ (\pi) \\ f_{1s} \\ 0 \\ f_{3s} \end{pmatrix} + \begin{pmatrix} x_0 \\ x_1 \\ x_2 \\ x_3 \\ x_4 \\ x_5 \end{pmatrix} \cdot exp(\lambda t), \qquad \text{(S3)}$$

and find the following equation for eigenvalues:

$$det \begin{bmatrix} k_1 - \lambda & k_2 m & k_3 m & k_4 & k_4 & k_4 \cdot m \\ k_2 m & k_1 - \lambda & -k_3 \cdot m & k_4 & -k_4 & k_4 \cdot m \\ -k_5 \cdot m & k_5 \cdot m & -2k_2 m - \lambda & 0 & -k_6 m & 0 \\ -k_7 & -k_7 & 0 & -k_8 - \lambda & 0 & -k_9 \\ -k_{10} & k_{10} & 0 & 0 & -k_8 - \lambda & 0 \\ -k_{11} & -k_{11} & 0 & \frac{-k_9}{2} & 0 & -k_8 - \lambda \end{bmatrix} \equiv 0. \qquad \text{(S4)}$$

Here

$$k_1 = \frac{\alpha_1}{1+\Delta^2} f_{1s} - \alpha_2 + A, k_2 = \frac{\alpha_1}{1+\Delta^2} f_{3s} + A, k_3 = \frac{\alpha_1 \Delta}{1+\Delta^2} f_{3s} E_s, k_4 = \frac{\alpha_1}{1+\Delta^2} E_s,$$

$$k_5 = \frac{2\alpha_1\Delta}{1+\Delta^2}\frac{f_{3s}}{E_s}, k_6 = \frac{2\alpha_1\Delta}{1+\Delta^2}, k_7 = \frac{2\chi\cdot\varepsilon\cdot E_s}{1+\Delta^2}(f_{1s} + f_{3s}\cdot m), \quad \text{(S5)}$$

$$k_8 = a_1 = \varepsilon\cdot\left(1 + \frac{I_p}{2} + \frac{2\chi E_s^2}{1+\Delta^2}\right), k_9 = \frac{2\varepsilon\cdot\chi E_s^2}{1+\Delta^2} m,$$

$$k_{10} = \frac{\chi\cdot\varepsilon\cdot E_s f_{1s}}{1+\Delta^2}, k_{11} = \frac{\chi\cdot\varepsilon\cdot E_s}{1+\Delta^2}(f_{1s}\cdot m + 2f_{3s}).$$

As a result, Eq. (SE4) takes the following form:

$$(2\lambda^3 + c_1\lambda^2 + c_2\lambda + c_3)\cdot(\lambda^3 + d_1\lambda^2 + d_2\lambda + d_3) \equiv 0. \quad \text{(S6)}$$

Here

$$c_1 = 4k_8 - 2k_1 - 2k_2 m,$$

$$c_2 = 2k_8^2 - 4k_8(k_1 + k_2) - \left(k_9^2 - 4k_4(k_7 + k_{11}m)\right),$$

$$c_3 = (k_9^2 - 2k_8^2)(k_1 + k_2 m) - 2k_4k_9(2k_{11} + k_7 m) + 4k_4k_8(k_7 + k_{11}m).$$

$$d_1 = k_8 - k_1 + 3k_2 m, \quad \text{(S7)}$$

$$d_2 = 2k_2^2 + k_2 m(3k_8 - 2k_1) + 2k_3k_5 - k_1k_8 m + 2k_4k_{10}m,$$

$$d_3 = 2k_8(k_2^2 - k_1k_2 m + k_3k_5) + 2k_{10}(2k_2k_4 m - k_3k_6).$$

For the following parameters: $\alpha_1 = 5.06, \varepsilon = 0.6\cdot 10^{-5}, \alpha_2 = 2.1, \Delta = 0.015$, $\chi = 2.3, \gamma = 2\cdot 10^{-6}, \delta = 1$ and $a = 0$ (no injected signal), the eigenvalue splits in two branches:

$$(I)\ \lambda_0 = R_0,\ \lambda_{1,2} = R_1 \pm i\Omega_{1,2}, \qquad (II)\ \lambda_3 = R_2, \qquad \lambda_{4,5} = R_3 \pm i\Omega_{3,4}$$

$$R_0 < 0,\ R_1 < 0, R_2 < 0\ (\Delta\varphi = 0, \pi), \quad \text{(S8)}$$

$$R_3 < 0\ (\Delta\varphi = 0), R_3 > 0\ (\Delta\varphi = \pi).$$

All eigenvalues with negative real part, e. g. $\lambda_0 - \lambda_3$, are shown in Fig. S2.

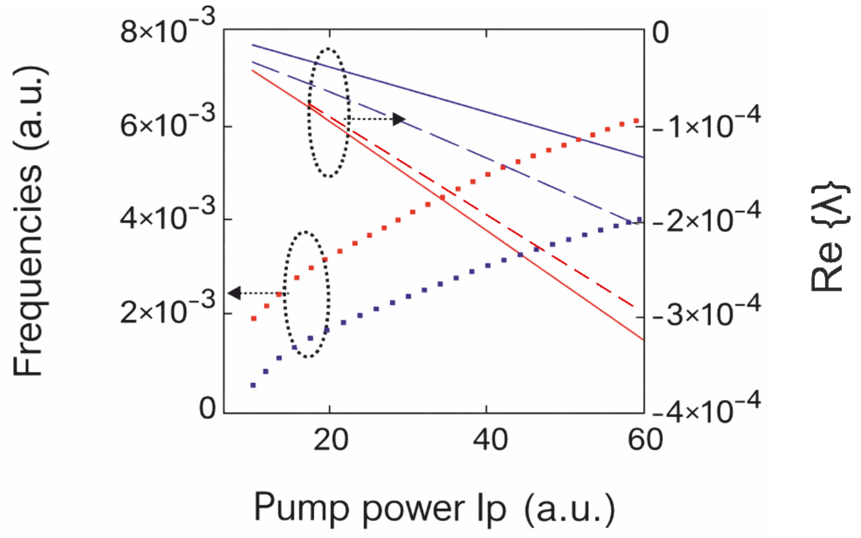

Fig. S2 Steady-state stability analysis of E-doped fibre laser without CW signal in the laser cavity. Eigenvalues with negative real parts as a function of the pump power I_p, $\Delta\varphi = 0$ (red lines), $\Delta\varphi = \pi$ (blue lines); $\Omega_{1,2}$ (dotted lines); R_1(solid lines); R_0 (dashed lines).
