# Supplementary material for: Nonlinear synchronization through vector subharmonic entrainment
Source: Commun Phys. 2026 Jan 21;9(1):71. doi: 10.1038/s42005-026-02509-7 (PMC12923357; doi:10.1038/s42005-026-02509-7)
Supplement: Supplementary file 3 — Reporting summary [file 42005_2026_2509_MOESM3_ESM.pdf]

nature research

Corresponding author(s): Dmitrii Stoliarov (d.stoliarov@aston.ac.uk)

Lasing Reporting Summary

Nature Research wishes to improve the reproducibility of the work that we publish. This form is intended for publication with all accepted papers reporting claims of lasing and provides structure for consistency and transparency in reporting. Some list items might not apply to an individual manuscript, but all fields must be completed for clarity.

For further information on Nature Research policies, including our data availability policy, see Authors & Referees.

~ Experimental design

Please check: are the following details reported in the manuscript?

1. Threshold

Plots of device output power versus pump power over a wide range of values indicating a clear threshold

☐ Yes
☒ No

All measurements used a 976 nm pump diode operated at 72 mW, chosen well above the lasing and mode-locking thresholds to ensure stable mode-locked and partially mode-locked operation. Because this study focuses on synchronization dynamics in an established fiber laser operating far above threshold, we did not include a full output-power–versus–pump-power curve; instead, we set the pump above the mode-locking threshold and confirmed entry into the stable QSML regime by monitoring the optical spectrum and photodetector signal while increasing the pump.

2. Linewidth narrowing

Plots of spectral power density for the emission at pump powers below, around, and above the lasing threshold, indicating a clear linewidth narrowing at threshold

☐ Yes
☒ No

Not provided / not applicable. This study investigates vector subharmonic entrainment in a passively mode-locked fiber laser operated well above the lasing and mode-locking thresholds; therefore, we did not record spectra below, around, and above the lasing threshold to demonstrate linewidth narrowing. Optical spectra are presented only in the regimes relevant to the investigation and were measured using an optical spectrum analyzer (OSA) AQ6317B (Yokogawa)

Resolution of the spectrometer used to make spectral measurements

☒ Yes
☐ No

Methods section provides the spectral resolution.

3. Coherent emission

Measurements of the coherence and/or polarization of the emission

☒ Yes
☐ No

We characterized the polarization dependence of the emission using polarization-resolved measurements , and tracked how the output changes during entrainment . We measured the polarization dynamics of the laser emission using a fast polarimeter (PM1000-XL-FA-N20 D, Novoptel) with a sampling rate of 100 MS/s. The polarimeter recorded the time evolution of the Stokes parameters, the powers in the orthogonal polarization components , and the total power; the phase difference was then recalculated from the measured Stokes parameters as described in Methods. The observed polarization dynamics support the vector-coupling mechanism underlying VSHE.

4. Beam spatial profile

Image and/or measurement of the spatial shape and profile of the emission, showing a well-defined beam above threshold

☐ Yes
☒ No

The laser output was delivered through single-mode fiber, so the emission is inherently in the fundamental spatial mode with a well-defined beam profile above threshold. Because the work focuses on polarization-mediated synchronization dynamics rather than free-space beam characterization, we did not include a separate camera image or quantitative beam-profile measurement.

5. Operating conditions

Description of the laser and pumping conditions
Continuous-wave, pulsed, temperature of operation

☒ Yes
☐ No

A full description of the experimental setup, including a schematic, components, fiber lengths, and pump powers, is provided in the Supplementary Information.

Threshold values provided as density values (e.g. W cm^{-2} or J cm^{-2}) taking into account the area of the device

☐ Yes
☒ No

We did not report the threshold as a density value because this is not standard practice for fiber lasers; thresholds are typically specified in terms of launched pump power.

6. Alternative explanations

Reasoning as to why alternative explanations have been ruled out as responsible for the emission characteristics

e.g. amplified spontaneous, directional scattering; modification of fluorescence spectrum by the cavity

☐ Yes
☒ No

Alternative explanations were ruled out because the laser shows clear, repeatable mode-locking/QSML signatures in the photodetector/RF measurements.

7. Theoretical analysis

Theoretical analysis that ensures that the experimental values measured are realistic and reasonable

e.g. laser threshold, linewidth, cavity gain-loss, efficiency

☒ Yes
☐ No

The theoretical model uses experimentally realistic cavity and polarization parameters and reproduces the observed entrainment behavior, including the measured locking ratios and polarization evolution.

8. Statistics

Number of devices fabricated and tested

☐ Yes
☒ No

Since this work builds on a well-established passively mode-locked fiber ring cavity architecture, we did not treat the number of fabricated devices as a key consideration; the experiments were performed using a single standard ring-cavity laser system, with repeated measurements to confirm reproducibility.

Statistical analysis of the device performance and lifetime (time to failure)

☐ Yes
☒ No

Device lifetime or time-to-failure statistics were not measured, as long-term reliability testing was outside the scope of this study, which focuses on synchronization dynamics in an established fiber ring laser operated under stable laboratory conditions.
